# Preclinical Pharmacological Evaluation of Sacituzumab Govitecan (IMMU-132) in TROP2-Positive Colorectal Liver Metastasis Models

**DOI:** 10.3390/ph19081163

**Published:** 2026-07-25

**Authors:** Weili Zhang, Ruowei Wang, Yingting Situ, Weifeng Wang, Jianhong Peng, Zhenhai Lu

**Affiliations:** Department of Colorectal Surgery, State Key Laboratory of Oncology in South China, Collaborative Innovation Center for Cancer Medicine, Sun Yat-sen University Cancer Center, 651 Dongfeng Road East, Guangzhou 510060, China

**Keywords:** sacituzumab govitecan, IMMU-132, TROP2, TACSTD2, antibody–drug conjugate, colorectal liver metastasis, patient-derived organoids

## Abstract

**Background/Objectives**: Sacituzumab govitecan (SG, IMMU-132) is a TROP2-directed antibody–drug conjugate carrying SN-38. Patients with colorectal liver metastasis (CRLM) still have limited treatment options after first-line systemic therapy, and the preclinical pharmacological value of TROP2-directed SN-38 delivery in CRLM remains insufficiently defined. **Methods**: Public single-cell RNA-sequencing data were reanalyzed to explore the distribution of TACSTD2/TROP2-positive epithelial-associated cells in adjacent normal tissues, primary colorectal cancer, and CRLM. The prognostic relevance of TACSTD2 was evaluated using the Kaplan–Meier Plotter database. TROP2-knockdown and TROP2-overexpressing colorectal cancer models were used to assess IMMU-132 response in vitro and in vivo. Pharmacological antitumor activity was further evaluated using subcutaneous xenografts, syngeneic intrasplenic liver metastasis models, and CRLM patient-derived organoids (PDOs). Transcriptomic profiling and γ-H2AX immunofluorescence were used to explore treatment-associated molecular changes. **Results**: At the patient/sample level, TACSTD2/TROP2-positive epithelial-associated cells showed numerically higher proportions in primary colorectal tumors and liver metastases than in adjacent normal tissues, and high TACSTD2 expression was associated with inferior overall survival in a public survival database. TROP2 knockdown attenuated IMMU-132-induced growth inhibition, apoptosis, and suppression of colony formation. In vivo, IMMU-132 suppressed colorectal tumor growth and reduced liver metastatic burden, with more evident activity in human TROP2-overexpressing models. In a limited exploratory CRLM PDO cohort established after first-line therapy, liver metastasis-derived PDOs showed lower normalized AUC values than primary tumor-derived PDOs. Transcriptomic analysis and representative γ-H2AX immunofluorescence suggested that IMMU-132 treatment was associated with changes in adhesion/cytoskeletal-related pathways, Wnt/cancer-associated transcriptional programs, and DNA damage-associated signals. **Conclusions**: These in vitro, in vivo, and PDO-based findings support further preclinical pharmacological evaluation of TROP2-directed SN-38 delivery by IMMU-132 in biomarker-annotated CRLM models, particularly in the post-first-line systemic therapy setting.

## 1. Introduction

Colorectal cancer is one of the most commonly diagnosed malignancies worldwide and remains a major cause of cancer-related mortality [1]. The liver is the most frequent site of distant metastasis in colorectal cancer, and approximately one-quarter of patients develop colorectal liver metastasis (CRLM) during the course of their disease [2]. In recent years, advances in multidisciplinary management, hepatic resection, locoregional treatment, and systemic therapy have improved the long-term outcomes of selected patients with CRLM. However, for patients with initially unresectable disease, high tumor burden, or progression after prior treatment, current chemotherapy-based regimens combined with targeted agents still face major challenges, including limited response durability, acquired resistance, and insufficient therapeutic options [2,3]. Therefore, the identification of novel therapeutic targets and more efficient drug-delivery strategies remains an important unmet need in CRLM treatment.

Antibody–drug conjugates (ADCs) represent an emerging class of anticancer agents that combine the target specificity of monoclonal antibodies with the cytotoxic potency of chemotherapeutic payloads, thereby enabling more selective delivery of cytotoxic drugs to antigen-expressing tumor cells [4]. Sacituzumab govitecan (SG, IMMU-132) is a trophoblast cell-surface antigen 2 (TROP2)-directed ADC composed of an anti-TROP2 antibody, a hydrolysable linker, and SN-38, the active metabolite of irinotecan and a topoisomerase I inhibitor [5,6]. Because SG uses a hydrolysable linker and releases membrane-permeable SN-38, it may exert bystander killing in tumors with heterogeneous TROP2 expression, a feature that may be relevant to CRLM where tumor-cell states and the liver metastatic microenvironment are heterogeneous [7,8]. Previous clinical studies have demonstrated the antitumor activity of SG in triple-negative breast cancer and several advanced epithelial malignancies [9,10]. Nevertheless, SG monotherapy has shown only modest activity in unselected metastatic colorectal cancer, highlighting the need to explore more appropriate disease contexts and biomarker-selection strategies [9].

TROP2, encoded by TACSTD2, is a transmembrane glycoprotein that is frequently upregulated in epithelial malignancies and has been implicated in tumor-cell proliferation, invasion, metastasis, and unfavorable prognosis [11,12]. Prior studies have suggested TROP2 as a promising therapeutic target for ADC-based treatment in solid tumors [5,6]. In the context of colorectal cancer and CRLM, previous tissue-level and protein-level studies from our center have provided complementary support for the clinical and biological relevance of TROP2. In a large colon cancer tissue microarray study, TROP2 protein expression assessed by immunohistochemistry was associated with survival, disease recurrence, and liver metastasis [13]. In patients undergoing liver resection for colorectal liver oligometastases, TROP2 overexpression in liver metastatic lesions was associated with unfavorable postoperative outcomes [14]. More recently, TROP2-mediated glycolysis and H3K18 lactylation were shown to form a feedback regulatory loop that promotes colorectal cancer liver metastatic progression [15]. Independent colorectal cancer cohorts have also identified TROP2 as a negative prognostic marker and linked its expression to invasive and epithelial–mesenchymal transition-associated features [16,17]. However, the single-cell distribution of TACSTD2/TROP2-positive epithelial-associated cells within CRLM lesions and whether TROP2 expression influences SG/IMMU-132 response in CRLM models remain to be further clarified.

In the present study, we hypothesized that TACSTD2/TROP2-positive epithelial-associated tumor cells may represent a pharmacologically targetable population in CRLM and that TROP2 expression may be associated with SG/IMMU-132 response. To test this hypothesis, we first reanalyzed public single-cell RNA-sequencing data to explore the distribution of TACSTD2/TROP2-positive epithelial-associated cells across adjacent normal tissues, primary colorectal tumors, and colorectal liver metastases, and evaluated the prognostic relevance of TACSTD2 expression using a public survival database. We then systematically investigated the preclinical pharmacological activity of SG/IMMU-132 in colorectal cancer and CRLM using TROP2-knockdown and TROP2-overexpressing cell models, subcutaneous xenografts, syngeneic intrasplenic liver metastasis models, and patient-derived organoids (PDOs). Finally, transcriptomic profiling and γ-H2AX immunofluorescence were used to explore treatment-associated molecular changes. Together, this study aimed to provide a preclinical pharmacological rationale for further evaluation of TROP2-directed SN-38 delivery and potential patient-selection strategies in CRLM.

## 2. Results

### 2.1. Single-Cell Transcriptomic Reanalysis Suggests TACSTD2/TROP2-Positive Epithelial-Associated Cells in Tumor-Associated Regions of Colorectal Liver Metastasis

**Figure 1:**
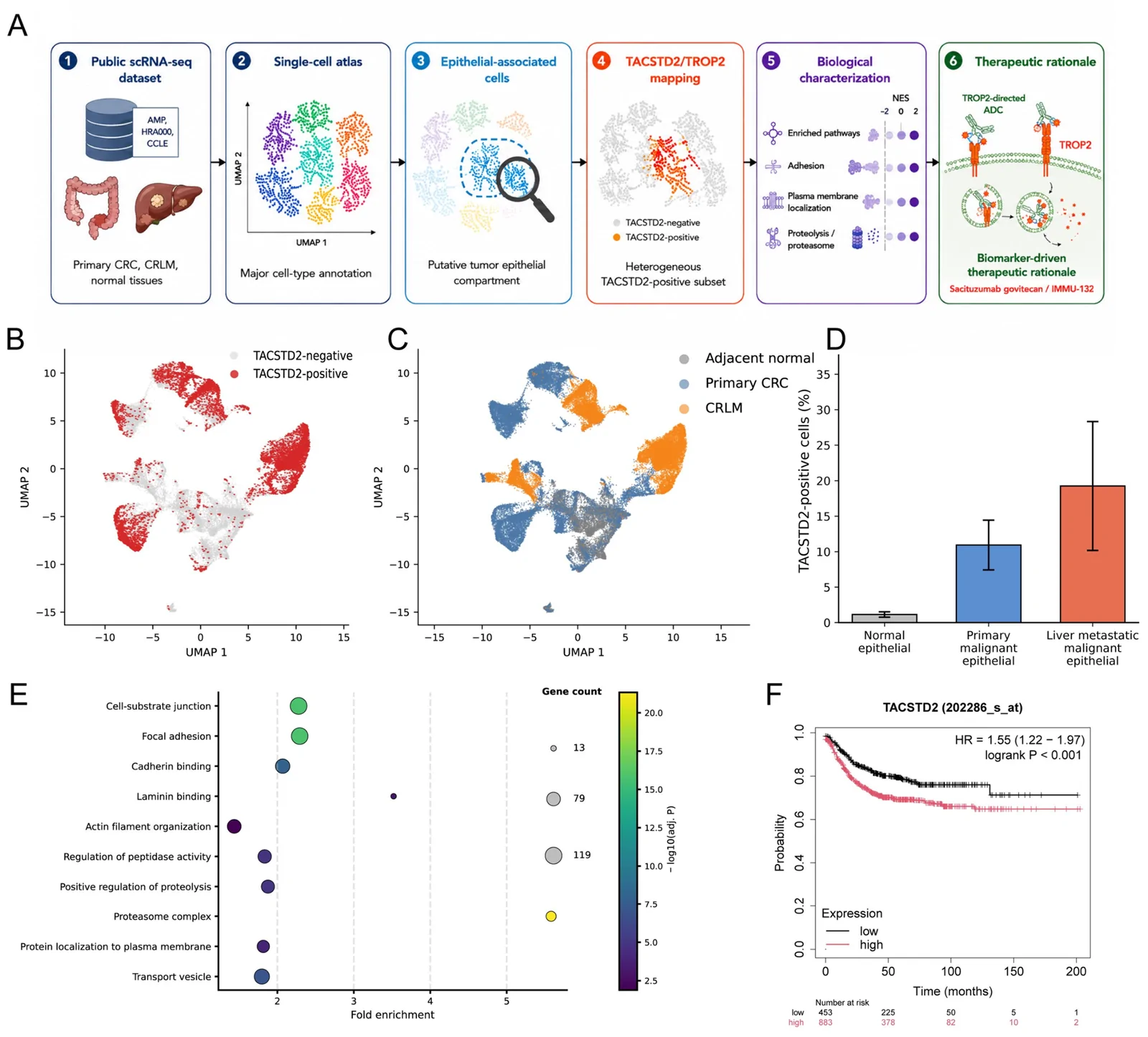
Exploratory single-cell transcriptomic reanalysis of TACSTD2/TROP2-positive epithelial-associated cells in colorectal cancer and colorectal liver metastasis. (**A**). Schematic overview of the single-cell transcriptomic analysis workflow, including public scRNA-seq dataset collection, major cell-type annotation, epithelial-associated cell selection, TACSTD2/TROP2 mapping, biological characterization, and therapeutic rationale for TROP2-directed ADC treatment. (**B**). UMAP visualization showing TACSTD2-positive and TACSTD2-negative cells within the epithelial-associated compartment. (**C**). UMAP projection of epithelial-associated cells colored by tissue origin, including adjacent normal tissue, primary colorectal cancer, and colorectal liver metastasis. TACSTD2-positive regions overlap mainly with primary CRC- and CRLM-derived epithelial-associated cells. (**D**). Patient/sample-level quantification of TACSTD2-positive epithelial-associated cell proportions across adjacent normal tissues (*n* = 6 patients/samples), primary colorectal tumors (*n* = 6 patients/samples), and liver metastases (*n* = 3 patients/samples). Bars show mean ± SEM. Because of the limited number of liver metastasis samples, this comparison is presented descriptively without formal patient-level statistical inference. (**E**). Functional enrichment analysis of TACSTD2-positive epithelial-associated cells. Dot size indicates gene count, and color indicates enrichment significance. (**F**). Kaplan–Meier overall survival analysis of colorectal cancer patients stratified by TACSTD2 expression using the Kaplan–Meier Plotter database. HR, hazard ratio. Data are presented as mean ± SEM where applicable. Abbreviations: ADC, antibody–drug conjugate; CRC, colorectal cancer; CRLM, colorectal liver metastasis; HR, hazard ratio; TACSTD2, tumor-associated calcium signal transducer 2; TROP2, trophoblast cell-surface antigen 2; UMAP, Uniform Manifold Approximation and Projection.

To establish a biomarker-based rationale for evaluating IMMU-132 in colorectal liver metastasis, we reanalyzed the public single-cell RNA-sequencing dataset GSE315534 [18], a whole-tumor scRNA-seq atlas comprising adjacent normal tissues, primary colorectal cancer, and colorectal liver metastases. The reanalyzed dataset included adjacent normal tissues from 6 patients/samples, primary colorectal tumors from 6 patients/samples, and liver metastases from 3 patients/samples. After major cell-type annotation, we extracted the epithelial-associated compartment for focused analysis. This compartment comprised 10,089 cells from adjacent normal tissues, 19,312 cells from primary colorectal tumors, and 16,999 cells from liver metastases. These epithelial-associated clusters expressed canonical epithelial markers, including EPCAM and multiple keratins, whereas limited cluster-specific non-epithelial marker signals were also observed, supporting their annotation as broad epithelial-associated rather than purified epithelial cells (Figure S1A,B). TACSTD2, the gene encoding TROP2, was then mapped within this epithelial-associated compartment (Figure 1A).

Within the epithelial-associated compartment, TACSTD2-positive cells showed a heterogeneous distribution and were mainly concentrated in tumor-associated epithelial regions rather than being uniformly distributed across all epithelial-associated cells (Figure 1B). Projection of epithelial-associated cells according to tissue origin further showed that the regions occupied by TACSTD2-positive cells overlapped substantially with cells derived from primary colorectal tumors and colorectal liver metastases, whereas they showed limited overlap with the major distribution of adjacent normal epithelial-associated cells (Figure 1C). To avoid cell-level pseudo-replication, we evaluated TACSTD2-positive proportions at the patient/sample level. At the sample level, TACSTD2-positive epithelial-associated cells showed numerically higher proportions in primary colorectal tumors and liver metastases than in adjacent normal tissues, with the highest median proportion observed in liver metastasis samples (Figure S1C; group-level summary shown in Figure 1D). Because only three liver metastasis samples were available, this comparison is presented descriptively without formal patient-level statistical inference. These findings suggest that the public scRNA-seq reanalysis provides an exploratory cellular localization signal for TACSTD2/TROP2-positive epithelial-associated cells in tumor-associated regions of primary and metastatic lesions.

To further characterize the biological features of TACSTD2-positive cells, we compared TACSTD2-positive and TACSTD2-negative epithelial-associated cells and performed functional enrichment analysis. TACSTD2-positive cells were enriched in processes related to cell–substrate junction, focal adhesion, cadherin binding, laminin binding, and protein localization to the plasma membrane (Figure 1E). These features may be related to adhesion- and membrane-localization-associated properties and provide a potential biological context for the binding and intracellular delivery of TROP2-targeted antibody–drug conjugates.

We also evaluated the prognostic relevance of TACSTD2 expression in colorectal cancer using the Kaplan–Meier Plotter online database. Patients with high TACSTD2 expression showed worse overall survival than those with low TACSTD2 expression (HR = 1.55, 95% CI 1.22–1.97; log-rank *p* < 0.001; Figure 1F). Collectively, the exploratory single-cell reanalysis and public survival analysis support the biological rationale for subsequent evaluation of the TROP2-directed ADC IMMU-132 in CRLM models, while requiring further validation in independent biomarker-annotated cohorts.

### 2.2. TROP2 Knockdown Attenuates IMMU-132-Induced Cytotoxicity in Colorectal Cancer Cells

**Figure 2:**
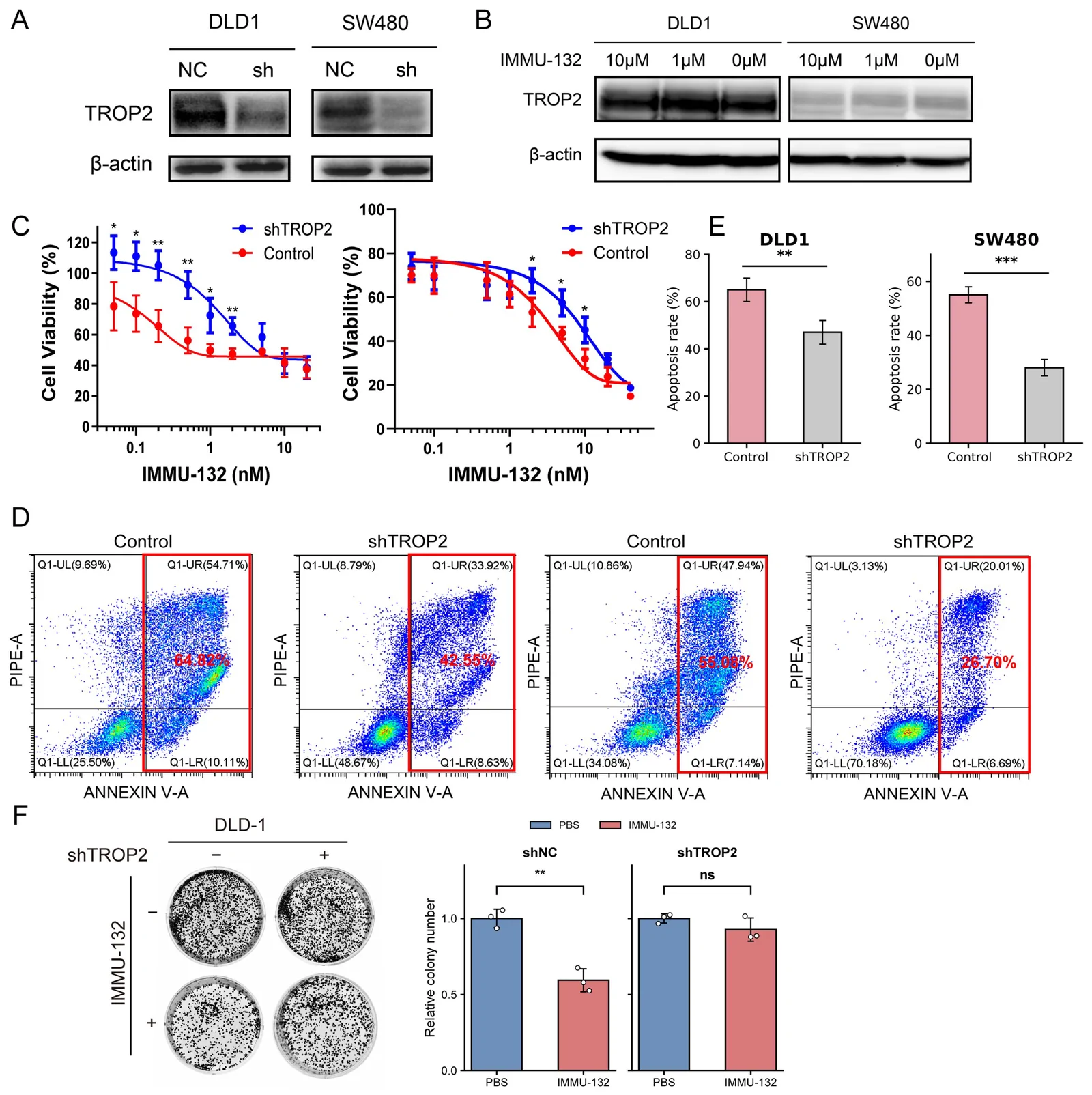
TROP2 knockdown attenuates IMMU-132-induced cytotoxicity in colorectal cancer cells. (**A**). Western blot analysis confirming TROP2 knockdown in DLD1 and SW480 cells. β-actin was used as the loading control. (**B**). Western blot analysis of TROP2 expression in DLD1 and SW480 cells treated with the indicated concentrations of IMMU-132. β-actin was used as the loading control. (**C**). CCK-8 cell viability assay of control and shTROP2 DLD1 and SW480 cells after treatment with serial concentrations of IMMU-132. Cell viability was normalized to the vehicle-treated control group. Experiments were performed with at least three independent biological replicates. (**D**). Representative Annexin V/PI flow cytometry plots showing apoptosis in control and shTROP2 DLD1 and SW480 cells after IMMU-132 treatment. (**E**). Quantification of apoptotic cells in DLD1 and SW480 cells after IMMU-132 treatment. Experiments were performed with at least three independent biological replicates. (**F**). Representative colony formation images and quantification of colony numbers in shNC and shTROP2 DLD1 cells treated with PBS or IMMU-132. Experiments were performed with at least three independent biological replicates. Data are presented as mean ± SEM. * *p* < 0.05, ** *p* < 0.01, *** *p* < 0.001. Abbreviations: IMMU-132, sacituzumab govitecan; NC, non-targeting shRNA negative control; PBS, phosphate-buffered saline; PI, propidium iodide; shNC, short hairpin negative control; shTROP2, short hairpin RNA targeting TROP2; TROP2, trophoblast cell-surface antigen 2. Red boxes in panel D highlight the Annexin V-positive/PI-positive upper-right quadrant, and the red percentages denote the total apoptotic fraction calculated as the sum of the Annexin V-positive quadrants. ns, not significant.

To determine whether TROP2 expression affects the response of colorectal cancer cells to IMMU-132, we generated stable TROP2-knockdown DLD1 and SW480 cell lines. Western blot analysis confirmed that TROP2 protein expression was markedly reduced in shTROP2 cells compared with the corresponding control cells (Figure 2A). In contrast, treatment with increasing concentrations of IMMU-132 did not substantially alter TROP2 protein expression in either DLD1 or SW480 cells (Figure 2B), suggesting that IMMU-132 response is more closely associated with baseline TROP2 expression than with drug-induced modulation of TROP2 expression.

CCK-8 cell viability assays showed that IMMU-132 inhibited the viability of DLD1 and SW480 cells in a dose-dependent manner. After TROP2 knockdown, the growth-inhibitory effect of IMMU-132 was attenuated, and shTROP2 cells retained higher cell viability across multiple drug concentrations (Figure 2C).

We next used Annexin V/PI double-staining flow cytometry to evaluate apoptosis after IMMU-132 treatment. IMMU-132-induced apoptosis was markedly reduced after TROP2 knockdown. In DLD1 cells, the apoptotic fraction decreased from 64.82% in control cells to 42.55% in shTROP2 cells; in SW480 cells, the apoptotic fraction decreased from 55.08% in control cells to 26.70% in shTROP2 cells (Figure 2D,E).

To further assess the effect of TROP2 knockdown on long-term proliferative capacity after IMMU-132 exposure, colony formation assays were performed in DLD1 cells. IMMU-132 markedly inhibited colony formation in control cells, whereas shTROP2 cells retained a higher colony-forming capacity after IMMU-132 treatment (Figure 2F). These findings indicate that TROP2 expression contributes to IMMU-132-mediated inhibition of cell viability, induction of apoptosis, and suppression of long-term proliferation, and that TROP2 knockdown attenuates the response of colorectal cancer cells to IMMU-132.

### 2.3. IMMU-132 Suppresses Colorectal Cancer Tumor Growth and Liver Metastatic Burden In Vivo

**Figure 3:**
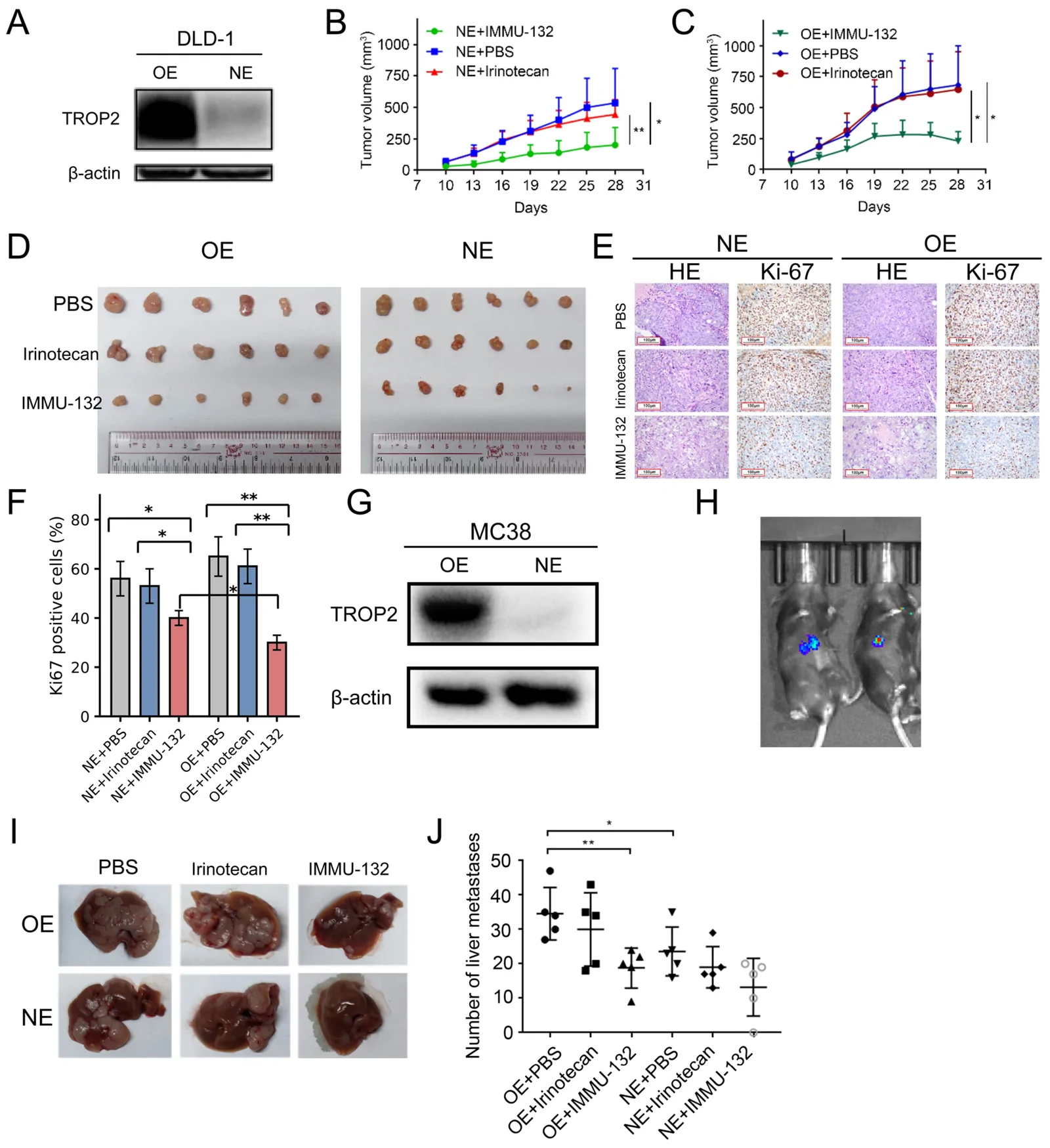
IMMU-132 suppresses colorectal cancer growth and liver metastatic burden in vivo. (**A**). Western blot analysis confirming TROP2 overexpression in DLD1 cells transduced with a human TACSTD2/TROP2-overexpressing vector (DLD1-hTROP2-OE) or negative control vector (DLD1-NE). β-actin was used as the loading control. (**B**,**C**). Tumor growth curves of DLD1-NE (**B**) and DLD1-hTROP2-OE (**C**) subcutaneous xenografts treated with PBS, irinotecan, or IMMU-132. Each treatment group included six mice. Irinotecan was included as an exploratory SN-38-related reference control under the dosing regimen used in this study. (**D**). Representative images of excised subcutaneous xenograft tumors from the indicated treatment groups. (**E**). Representative HE and Ki-67 immunohistochemistry images of xenograft tumor tissues from the indicated groups. (**F**). Quantification of Ki-67-positive cells in xenograft tumors. Ki-67-positive cells were quantified in representative microscopic fields by investigators blinded to treatment allocation where feasible. (**G**). Western blot analysis confirming TROP2 overexpression in human TACSTD2/TROP2-engineered MC38 cells (MC38-hTROP2-OE) and corresponding negative-control cells (MC38-NE). β-actin was used as the loading control. (**H**). Representative in vivo bioluminescence imaging after intrasplenic injection of luciferase-expressing MC38-NE or MC38-hTROP2-OE cells, confirming successful tumor establishment in the liver. (**I**). Representative gross liver images from the MC38 intrasplenic injection liver metastasis model after treatment with PBS, irinotecan, or IMMU-132. (**J**). Quantification of liver metastatic foci in the indicated groups. Each treatment group included five mice. Data are presented as mean ± SEM. * *p* < 0.05, ** *p* < 0.01. Abbreviations: HE, hematoxylin and eosin; hTROP2, human TROP2; IMMU-132, sacituzumab govitecan; IVIS, In Vivo Imaging System; NE, empty-vector negative control; OE, overexpression; PBS, phosphate-buffered saline; TROP2, trophoblast cell-surface antigen 2.

We next evaluated the antitumor activity of IMMU-132 in vivo. Stable human TROP2-overexpressing DLD1 cells were generated, and TROP2 overexpression in DLD1-hTROP2-OE cells was confirmed by Western blotting (Figure 3A). DLD1-NE and DLD1-hTROP2-OE cells were then used to establish subcutaneous xenograft models. In the DLD1-NE xenograft model, IMMU-132 treatment delayed tumor growth compared with PBS treatment under the dosing regimen used in this study, while irinotecan was included as an exploratory SN-38-related reference control (Figure 3B). Similar results were observed in the DLD1-hTROP2-OE model, in which tumor volume was lower in the IMMU-132 group than in the PBS group (Figure 3C). Consistent with the tumor growth curves, images of excised tumors showed smaller tumors in the IMMU-132-treated groups (Figure 3D).

Histological analysis further supported the in vivo antitumor activity of IMMU-132. HE staining showed reduced tumor cellularity after IMMU-132 treatment, and Ki-67 immunohistochemistry revealed decreased tumor-cell proliferation in the IMMU-132-treated groups (Figure 3E). Quantification of Ki-67-positive cells further showed that the proportion of Ki-67-positive cells was lower in the IMMU-132-treated groups, with a more pronounced reduction in human TROP2-overexpressing tumors (Figure 3F).

To further assess the preclinical pharmacological activity of IMMU-132 in colorectal liver metastasis, we established an engineered syngeneic intrasplenic liver metastasis model using luciferase-expressing MC38 cells. Human TROP2 overexpression in MC38-hTROP2-OE cells was verified by Western blotting, and in vivo bioluminescence imaging after intrasplenic injection confirmed successful tumor establishment in the liver (Figure 3G,H). MC38-NE and MC38-hTROP2-OE liver metastasis-bearing mice were then treated with PBS, irinotecan, or IMMU-132. At the experimental endpoint, gross liver images showed that IMMU-132 treatment reduced liver metastatic burden compared with PBS treatment (Figure 3I). Quantification of liver metastatic foci showed that IMMU-132 reduced the number of liver metastases, with a more evident inhibitory effect in the MC38-hTROP2-OE group than in the MC38-NE group (Figure 3J). These in vivo data suggest that IMMU-132 suppresses colorectal cancer tumor growth and liver metastasis formation, with more evident antitumor activity in human TROP2-overexpressing models.

### 2.4. IMMU-132 Inhibits Patient-Derived Colorectal Cancer Organoids, with Lower Normalized AUC Values in Liver Metastasis-Derived PDOs

**Figure 4:**
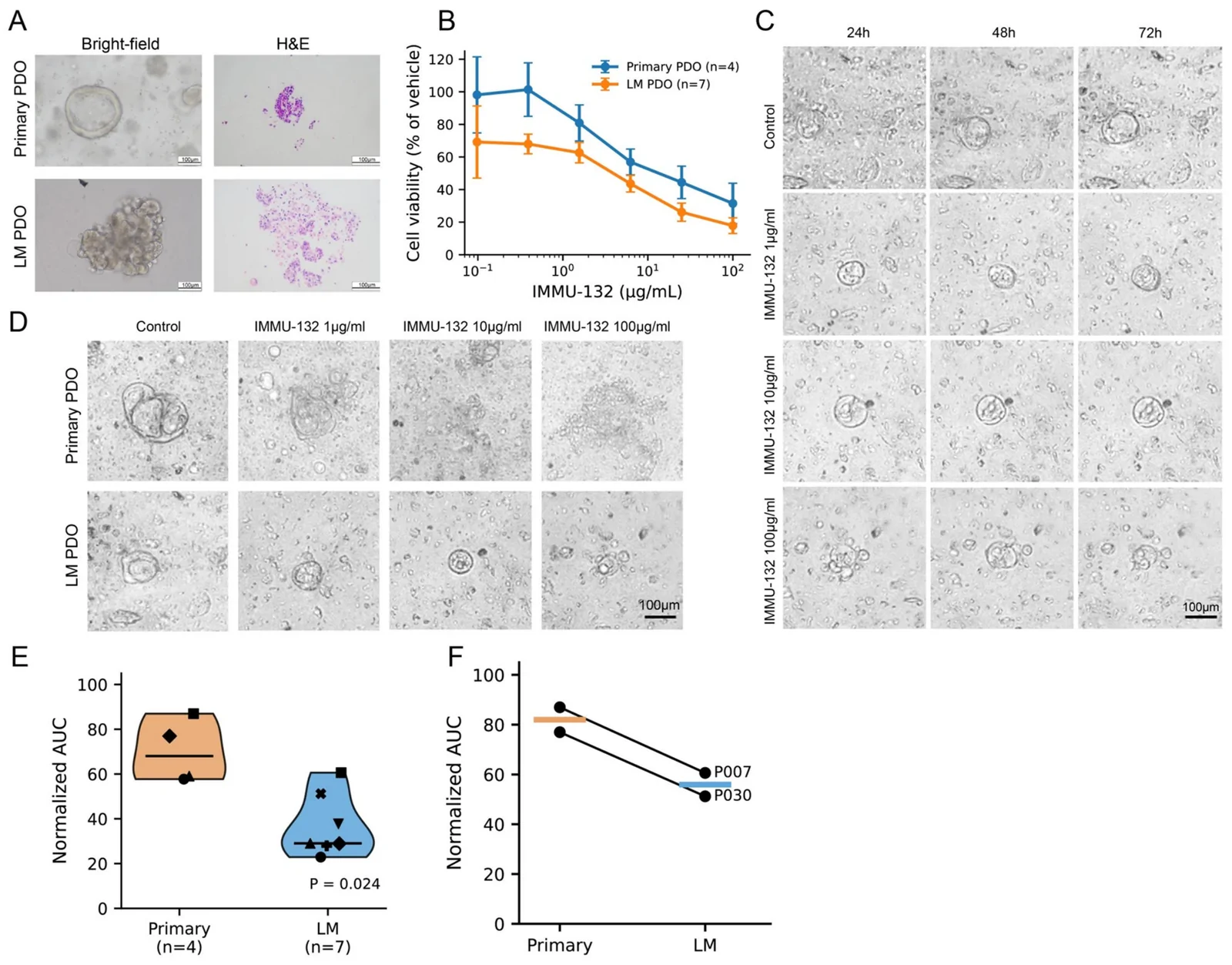
IMMU-132 inhibits patient-derived colorectal cancer organoids, with lower normalized AUC values in liver metastasis-derived PDOs in an exploratory cohort. (**A**). Representative bright-field and H&E staining images of patient-derived organoids established from primary colorectal tumors and liver metastatic lesions. Scale bars, 100 μm. (**B**). Mean dose–response curves of primary tumor-derived PDOs (*n* = 4) and liver metastasis-derived PDOs (*n* = 7) treated with serial concentrations of IMMU-132. Cell viability was determined by CellTiter-Glo assay and normalized to the vehicle-treated control group. Primary PDO, primary tumor-derived PDO; LM PDO, liver metastasis-derived PDO. (**C**). Representative time-course images of PDOs treated with vehicle or the indicated concentrations of IMMU-132 for 24 h, 48 h, and 72 h. Scale bar, 100 μm. (**D**). Representative 72 h morphology images of primary tumor-derived PDOs and liver metastasis-derived PDOs treated with vehicle or the indicated concentrations of IMMU-132. Scale bar, 100 μm. (**E**). Normalized AUC summary of IMMU-132 dose–response curves in primary tumor-derived PDOs (*n* = 4) and liver metastasis-derived PDOs (*n* = 7). Lower normalized AUC indicates stronger drug-induced inhibition. The comparison is presented as an exploratory analysis because of the limited PDO cohort size. (**F**). Matched comparison of normalized AUC values in primary tumor-derived and liver metastasis-derived PDOs from patients P007 and P030 (*n* = 2 matched pairs). PDOs were established from a CRLM cohort receiving first-line systemic therapy and undergoing resection of primary tumors or liver metastases. Abbreviations: AUC, area under the curve; CRLM, colorectal liver metastasis; HE, hematoxylin and eosin; IMMU-132, sacituzumab govitecan; LM, liver metastasis; PDO, patient-derived organoid.

To further determine the preclinical pharmacological value of IMMU-132 for CRLM treatment, we established a patient-derived organoid (PDO) cohort from patients with CRLM who had received first-line systemic therapy and underwent resection of primary tumors or liver metastatic lesions. Detailed clinical information and molecular background of this cohort are provided in Table S1. A total of 11 PDOs from 9 patients were included in the IMMU-132 drug-response analysis, comprising 7 liver metastasis-derived PDOs and 4 primary tumor-derived PDOs. Among them, patients P007 and P030 had matched PDOs derived from both the primary tumor and liver metastasis. Representative bright-field and H&E staining images showed that both primary tumor-derived and liver metastasis-derived samples formed typical organoid structures (Figure 4A).

We then used CellTiter-Glo viability assays to evaluate the response of PDOs to IMMU-132. In this limited exploratory PDO cohort, IMMU-132 generally reduced organoid viability across the tested concentration range, although individual PDOs showed heterogeneous response patterns at lower concentrations (Figure 4B). Individual dose–response curves for all IMMU-132-treated PDOs are provided in Figure S2. When stratified by lesion origin, liver metastasis-derived PDOs showed a stronger inhibitory trend than primary tumor-derived PDOs across multiple tested concentrations. Consistently, time-course morphological imaging showed progressive structural disruption of organoids with increasing treatment duration and drug concentration (Figure 4C). Representative 72 h images further showed that IMMU-132 reduced organoid density and disrupted organoid morphology in both primary tumor-derived and liver metastasis-derived PDOs, particularly at higher concentrations (Figure 4D).

To quantitatively summarize drug response across PDO samples, we calculated the normalized area under the dose–response curve (normalized AUC), with lower AUC values indicating stronger drug-induced inhibition. Liver metastasis-derived PDOs showed lower normalized AUC values than primary tumor-derived PDOs in this exploratory cohort (*p* = 0.024; Figure 4E). In addition, in the two matched PDO pairs from patients P007 and P030, liver metastasis-derived PDOs showed lower normalized AUC values than their corresponding primary tumor-derived PDOs (Figure 4F). An exploratory comparison of IMMU-132 and irinotecan responses in PDOs tested with both drugs is shown in Figure S3. These results indicate that IMMU-132 inhibits patient-derived colorectal cancer organoids and suggest that liver metastasis-derived PDOs may show greater drug response than primary tumor-derived PDOs, a finding that requires validation in larger biomarker-annotated CRLM PDO cohorts.

### 2.5. Transcriptomic Analysis Indicates IMMU-132-Associated Molecular Changes and Exploratory DNA Damage Signal

**Figure 5:**
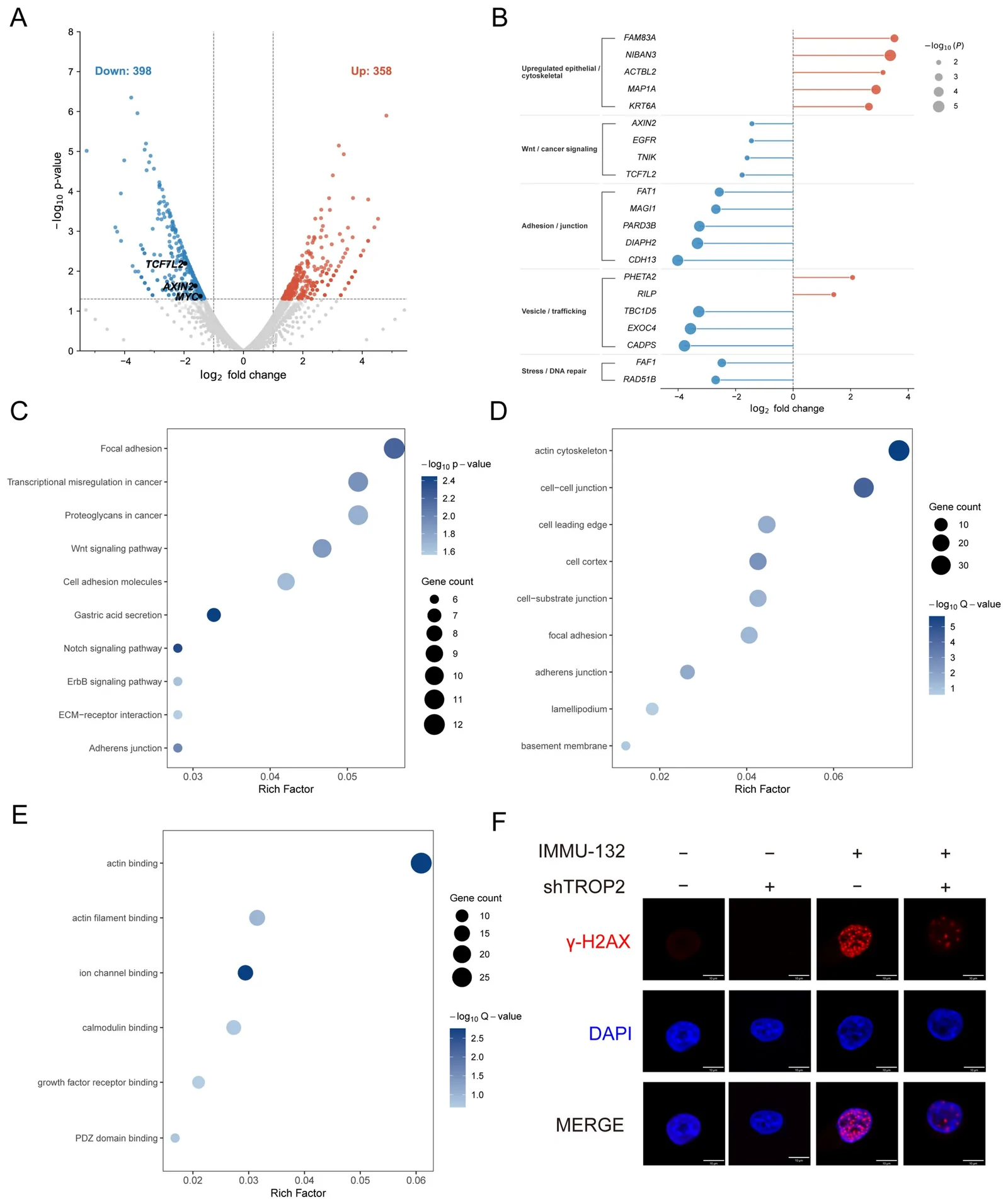
Transcriptomic profiling and γ-H2AX immunofluorescence show IMMU-132-associated molecular changes and exploratory DNA damage signal. (**A**). Volcano plot showing differentially expressed genes between IMMU-132-treated and control DLD1 cells. Red dots indicate upregulated genes, blue dots indicate downregulated genes, and gray dots indicate non-significant genes. (**B**). Selected differentially expressed genes grouped by biological function, including epithelial/cytoskeletal regulation, Wnt/cancer-associated transcriptional programs, adhesion/junction, vesicle trafficking, and stress/DNA repair. Dot size indicates −log10(P), and the *x*-axis indicates log2 fold change. (**C**). KEGG pathway enrichment analysis of differentially expressed genes after IMMU-132 treatment. Dot size indicates gene count, and color indicates −log10(*p* value). (**D**). GO cellular component enrichment analysis of differentially expressed genes. Dot size indicates gene count, and color indicates −log10(Q value). (**E**). GO molecular function enrichment analysis of differentially expressed genes. Dot size indicates gene count, and color indicates −log10(Q value). (**F**). Representative immunofluorescence images of γ-H2AX, DAPI, and merged channels in control and shTROP2 cells after vehicle or IMMU-132 treatment. Images are shown as exploratory supportive evidence and were not used for quantitative foci analysis. Scale bars, 10 μm. Abbreviations: DAPI, 4′,6-diamidino-2-phenylindole; GO, Gene Ontology; KEGG, Kyoto Encyclopedia of Genes and Genomes; IMMU-132, sacituzumab govitecan; shTROP2, short hairpin RNA targeting TROP2; TROP2, trophoblast cell-surface antigen 2.

To explore molecular changes in colorectal cancer cells after IMMU-132 treatment, we performed RNA sequencing of DLD1 cells treated with IMMU-132 or vehicle control. Differential expression analysis identified 358 upregulated and 398 downregulated genes (Figure 5A). Among the representative differentially expressed genes, epithelial/cytoskeletal-associated genes, including FAM83A, NIBAN3, ACTBL2, MAP1A, and KRT6A, were upregulated, whereas TCF7L2, AXIN2, MYC, and the DNA repair-associated gene RAD51B were downregulated (Figure 5A,B). The downregulation of TCF7L2, AXIN2, and MYC suggests that IMMU-132 treatment was associated with changes in Wnt/cancer-associated transcriptional programs, whereas changes in RAD51B and other DNA repair-related genes indicate that drug exposure may be accompanied by altered DNA damage response or repair capacity, consistent with the known activity of SN-38 as a topoisomerase I inhibitor.

Pathway enrichment analysis further showed that IMMU-132 treatment was associated with changes in cell adhesion, cytoskeletal organization, and cancer-related signaling pathways. KEGG enrichment analysis indicated that differentially expressed genes were mainly involved in focal adhesion, Wnt signaling pathway, cell adhesion molecules, ECM-receptor interaction, and adherens junction pathways (Figure 5C). GO cellular component analysis showed enrichment in actin cytoskeleton, cell–cell junction, cell–substrate junction, and focal adhesion-related structures (Figure 5D). GO molecular function analysis further suggested that actin binding, actin filament binding, growth factor receptor binding, and PDZ domain binding were affected (Figure 5E). These results indicate that IMMU-132 exposure was associated with treatment-related transcriptomic changes involving cytoskeletal organization, cell adhesion, and Wnt/cancer-associated transcriptional programs.

Notably, stress/DNA repair-related genes were also altered among the representative differentially expressed gene groups, suggesting that IMMU-132 exposure may be accompanied by DNA damage response changes. Given that the cytotoxic payload of IMMU-132 is SN-38, which induces replication stress and DNA damage through topoisomerase I inhibition, we further examined DNA damage-associated signal by γ-H2AX immunofluorescence. Representative immunofluorescence images showed that IMMU-132 treatment increased γ-H2AX foci in control cells, whereas the IMMU-132-associated γ-H2AX signal was reduced in shTROP2 cells (Figure 5F). This finding is consistent with reduced TROP2-mediated ADC binding, internalization, and/or SN-38 payload delivery after TROP2 knockdown, rather than evidence of a distinct TROP2-specific DNA damage pathway. Together with the transcriptomic changes, these representative data suggest that IMMU-132 treatment is associated with molecular changes involving adhesion/cytoskeletal-related pathways, Wnt/cancer-associated transcriptional programs, and DNA damage-associated signals.

## 3. Discussion

Colorectal liver metastasis remains a major therapeutic challenge in colorectal cancer, particularly for patients with initially unresectable disease, high metastatic burden, or progression after standard systemic therapy [2,3]. In the present study, we integrated public single-cell transcriptomic reanalysis, TROP2-manipulated cell models, subcutaneous xenograft and liver metastasis models, patient-derived organoids, and exploratory transcriptomic and DNA damage-associated analyses to evaluate the preclinical pharmacological activity and translational relevance of sacituzumab govitecan/IMMU-132 in colorectal liver metastasis. Our results suggest that TACSTD2/TROP2-positive epithelial-associated cells are detectable in tumor-associated regions of primary colorectal cancer and liver metastases; TROP2 expression is associated with IMMU-132 response in engineered models; and IMMU-132 suppresses colorectal tumor growth and liver metastatic burden in vivo. Importantly, all PDOs in this study were derived from CRLM specimens obtained after first-line therapy, providing an exploratory patient-derived model for evaluating TROP2-directed SN-38 delivery in the post-first-line setting.

Previous tissue-level and protein-level studies in colorectal cancer and CRLM provide important context for the present study. In a large colon cancer tissue microarray study from our center, TROP2 protein expression assessed by immunohistochemistry was associated with patient survival, disease recurrence, and liver metastasis [13]. In patients undergoing liver resection for colorectal liver oligometastases, TROP2 overexpression in liver metastatic lesions was associated with unfavorable postoperative outcomes [14]. More recently, a feedback regulatory loop between TROP2-mediated glycolysis and H3K18 lactylation was reported to promote metastatic progression of colorectal cancer [15]. These prior findings support the biological plausibility of TROP2-directed targeting in CRC/CRLM. However, they do not replace protein-level validation in the PDO cohort used in the present study, and the lack of systematic TROP2 protein quantification in our PDOs remains an important limitation.

The public single-cell RNA-sequencing reanalysis in this study should be viewed as an exploratory cellular localization analysis rather than definitive standalone evidence for the clinical relevance of TROP2. At the patient/sample level, TACSTD2-positive epithelial-associated cells showed numerically higher proportions in primary tumors and liver metastases than in adjacent normal tissues, but the liver metastasis group included only three samples. Therefore, we present this result descriptively and avoid cell-level statistical inference to reduce the risk of pseudo-replication. This single-cell signal complements, but does not replace, previous tissue-level and protein-level evidence. From a pharmacological perspective, the localization of TACSTD2/TROP2-positive cells within tumor-associated epithelial regions remains relevant because ADC activity depends on the presence of target antigen on tumor-cell populations and on the accessibility of antibody binding, internalization, and payload delivery.

Functional experiments further supported an association between TROP2 expression and IMMU-132 response. TROP2 knockdown attenuated IMMU-132-induced growth inhibition, apoptosis, and suppression of colony formation in colorectal cancer cells, whereas in vivo experiments showed antitumor responses to IMMU-132 in human TROP2-overexpressing models. These results are consistent with the basic pharmacology of a TROP2-directed ADC, in which antibody-mediated targeting facilitates delivery of the cytotoxic payload to TROP2-expressing tumor cells. However, IMMU-132 activity should not be interpreted as completely dependent on TROP2 expression. Baseline endogenous TROP2 expression, intratumoral SN-38 release, bystander effects, and model-specific pharmacokinetics may all contribute to activity. Because SG/IMMU-132 uses a hydrolysable linker and releases membrane-permeable SN-38, bystander killing may be particularly relevant in heterogeneous tumors and liver metastatic lesions [7,8].

Our findings are generally consistent with previous preclinical studies of IMMU-132 in other solid tumors. Early studies demonstrated that sacituzumab govitecan is an ADC composed of a humanized anti-TROP2 antibody conjugated to SN-38 through a hydrolysable linker, retaining tumor-binding activity and showing antitumor efficacy in multiple epithelial cancer xenograft models, including pancreatic, gastric, lung, breast, and colorectal cancer models [5,6,7]. Compared with these prior studies, our work extends the preclinical pharmacological evidence for IMMU-132 into the specific disease context of CRLM and adds several layers of CRLM-relevant biological and translational evidence, including exploratory single-cell localization of TACSTD2/TROP2-positive epithelial-associated cells, an intrasplenic liver metastasis model, and CRLM patient-derived organoids. Because liver metastases may differ from primary tumors in cellular composition, microenvironmental context, and drug response, these models are important for evaluating the pharmacological activity of IMMU-132 in CRLM.

From a pharmacological and translational perspective, sacituzumab govitecan has demonstrated the feasibility of TROP2-directed SN-38 delivery in multiple epithelial malignancies. In heavily pretreated metastatic triple-negative breast cancer, early studies showed clear antitumor activity of sacituzumab govitecan [19], and the phase III ASCENT trial subsequently demonstrated significant improvements in progression-free and overall survival compared with single-agent chemotherapy [10]. In metastatic urothelial carcinoma, the TROPHY-U-01 study also showed clinical activity of sacituzumab govitecan in patients who had progressed after platinum-based chemotherapy and immune checkpoint inhibitors [20]. However, the activity of sacituzumab govitecan monotherapy in unselected metastatic colorectal cancer has been modest. In the IMMU-132-01 basket trial, the colorectal cancer cohort had an objective response rate of only 3.2%, with a median progression-free survival of 3.9 months and a median overall survival of 14.2 months [9]. This discrepancy highlights the need for better biomarker selection and disease-context-specific evaluation when exploring TROP2-directed ADCs in colorectal cancer.

The PDO results provide an exploratory translational bridge between cell-line models and clinically relevant tumor specimens. PDOs can retain, to a certain extent, the histological, genomic, and drug-response features of patient tumors and have been increasingly used to model therapeutic responses in colorectal cancer and metastatic gastrointestinal cancers [21,22,23]. In the present study, all CRLM PDOs were established from specimens obtained after first-line therapy, making them representative of a clinically relevant post-first-line setting in which subsequent treatment options are needed. IMMU-132 generally inhibited organoid viability across the tested concentration range, and liver metastasis-derived PDOs showed lower normalized AUC values than primary tumor-derived PDOs in this limited cohort. A possible explanation is that treatment selection and the liver metastatic state may retain or enrich tumor-cell programs associated with TROP2 expression, altered adhesion state, cell-surface antigen accessibility, or ADC uptake. However, because systematic TROP2 protein expression, cell-surface TROP2 levels, and ADC internalization were not quantified across all PDOs, this observation should be regarded as hypothesis-generating and requires validation in larger biomarker-annotated CRLM PDO cohorts.

Transcriptomic analysis and γ-H2AX immunofluorescence provided additional molecular context but should be interpreted cautiously. IMMU-132 consists of a humanized anti-TROP2 antibody conjugated to SN-38 through a hydrolysable linker, and SN-38 is the active metabolite of irinotecan and a potent topoisomerase I inhibitor capable of inducing replication stress and DNA damage [5,6,7,24,25]. Against this pharmacological background, differentially expressed genes after IMMU-132 treatment were associated with focal adhesion, cell adhesion molecules, ECM-receptor interaction, adherens junction, cytoskeletal organization, and Wnt/cancer-associated transcriptional programs. The downregulation of Wnt/cancer-associated transcriptional genes, including TCF7L2, AXIN2, and MYC, suggests treatment-associated changes in tumor-related transcriptional states rather than a proven causal mechanism [26,27]. Similarly, representative γ-H2AX immunofluorescence suggested increased DNA damage-associated signal after IMMU-132 exposure, and the attenuation of this signal in shTROP2 cells is consistent with reduced TROP2-mediated ADC binding, internalization, and/or payload delivery [28]. Because γ-H2AX foci were not quantitatively analyzed and key RNA-seq findings were not independently validated by qPCR or Western blotting, these data should be regarded as exploratory supportive analyses.

Several limitations should be acknowledged. First, the single-cell analysis was based on a public dataset and was interpreted descriptively at the patient/sample level because of the limited number of liver metastasis samples. Independent CRLM cohorts, ideally combined with immunohistochemistry or spatial transcriptomic profiling, are needed to confirm the protein-level and spatial localization of TROP2 in tumor cells. Second, although we established PDOs from both primary tumors and liver metastases, the overall sample size remains limited, especially for matched primary tumor-liver metastasis PDO pairs. Third, the MC38 syngeneic liver metastasis model used an engineered human TACSTD2/TROP2-overexpression system. This model enabled evaluation of IMMU-132 activity in a TROP2-expressing liver metastasis model under an immunocompetent background, but it cannot fully recapitulate the natural heterogeneity of TROP2 expression in patient-derived CRLM. In addition, because MC38 is of murine origin whereas SG/IMMU-132 contains a humanized antibody backbone, repeated administration may induce anti-drug or anti-species antibody responses that could alter exposure, pharmacokinetics, or antibody activity. Fourth, the irinotecan arm was included only as an exploratory SN-38-related reference control under the dosing regimen used in this study and should not be interpreted as a conventional irinotecan efficacy comparator. Fifth, although RNA sequencing and γ-H2AX immunofluorescence suggested treatment-associated transcriptomic changes and DNA damage-associated signals, additional experiments, including ADC uptake assays, intracellular SN-38 quantification, quantitative γ-H2AX foci analysis, and validation of key genes, are needed. Finally, this is a preclinical study and cannot directly establish the clinical efficacy of IMMU-132 in patients with CRLM.

## 4. Materials and Methods

### 4.1. Single-Cell RNA-Sequencing Analysis

Single-cell RNA-sequencing data from GSE315534, generated in a recent study of colorectal cancer liver metastasis that profiled primary colorectal tumors, adjacent normal tissues, and paired liver metastases [18], were downloaded from the Gene Expression Omnibus as processed data and analyzed using Seurat (v5.3.1) in R (v4.4.2). The reanalyzed dataset included adjacent normal tissues from 6 patients/samples, primary colorectal tumors from 6 patients/samples, and liver metastases from 3 patients/samples. Quality-control filtering removed cells with fewer than 200 or more than 5000 detected genes and cells with mitochondrial gene percentages exceeding 20%. Data were log-normalized and scaled, and principal component analysis was performed using the top 2000 variable genes. Uniform Manifold Approximation and Projection (UMAP) was used for dimensionality reduction, and cells were clustered using the Louvain algorithm at a resolution of 0.5.

Major cell types were annotated according to canonical marker gene expression. The epithelial-associated compartment was extracted for focused analysis of TACSTD2, the gene encoding TROP2. Copy number inference was performed using CopyKAT (version 1.1.0) to help distinguish putative malignant epithelial-associated cells from normal epithelial-associated cells, together with canonical epithelial marker expression and tissue origin. TACSTD2 expression was visualized on UMAP embeddings and summarized across tissue origins. TACSTD2 positivity was defined as detectable normalized TACSTD2 expression. To avoid cell-level pseudo-replication, the proportion of TACSTD2-positive epithelial-associated cells was calculated at the patient/sample level. Because only three liver metastasis samples were available, sample-level comparisons were presented descriptively without formal patient-level statistical inference.

To characterize the biological features of TACSTD2-positive epithelial-associated cells, cells were divided into TACSTD2-positive and TACSTD2-negative groups according to detectable TACSTD2 expression in the normalized expression matrix. Differentially expressed genes between the two groups were identified using a non-parametric rank-based test. Gene Ontology (GO) enrichment analysis was performed using the clusterProfiler (version 4.14.6) package.

### 4.2. Public Survival Analysis

The prognostic relevance of TACSTD2 expression in colorectal cancer was evaluated using the Kaplan–Meier Plotter online database. Patients were stratified into high- and low-expression groups according to the database default cutoff. Overall survival was compared between groups using the log-rank test, and hazard ratios with 95% confidence intervals were obtained from the online analysis output.

### 4.3. Cell Cultures and Treatment

Human colorectal cancer cell lines SW480 and DLD1 and the murine colorectal cancer cell line MC38 were obtained from the American Type Culture Collection (ATCC, Manassas, VA, USA). SW480 and DLD1 cells were used for in vitro functional experiments, DLD1 cells were used for subcutaneous xenograft experiments, and luciferase-expressing MC38 cells were used to establish the syngeneic intrasplenic liver metastasis model. Cells were cultured in RPMI-1640 medium (Gibco, Carlsbad, CA, USA) supplemented with 10% fetal bovine serum (Gibco) and 1% penicillin-streptomycin at 37 °C in a humidified incubator containing 5% CO_2_. Cells were treated with sacituzumab govitecan (IMMU-132), irinotecan, or PBS as indicated in the corresponding experiments.

### 4.4. Generation of TROP2-Knockdown and TROP2-Overexpressing Cell Lines

Stable TROP2-knockdown DLD1 and SW480 cells were generated using lentiviral vectors carrying short hairpin RNA (shRNA) sequences targeting TROP2. Cells transduced with a non-targeting shRNA vector were used as negative controls (NC). Stable human TROP2-overexpressing DLD1 cells were generated by lentiviral transduction with a human TACSTD2/TROP2 overexpression vector, and corresponding empty-vector-transduced cells were used as empty-vector negative controls (NE). For the syngeneic liver metastasis model, luciferase-expressing MC38 cells were engineered to overexpress human TACSTD2/TROP2 (MC38-hTROP2-OE), and empty-vector-transduced MC38 cells served as negative controls (MC38-NE). The pEZ-Lv217-TROP2 lentiviral vector and corresponding control vector were constructed and confirmed by GeneCopoeia (Guangzhou, China). TROP2 knockdown or overexpression was verified by Western blotting before subsequent experiments.

### 4.5. CCK-8 Assay

For the cell viability assay, colorectal cancer cells were seeded in 96-well plates at a density of 1 × 10^4^ cells per well in 100 μL complete culture medium. The following day, the culture medium was replaced with 100 μL fresh medium containing IMMU-132 at concentrations of 0, 0.05, 0.1, 0.2, 0.5, 1, 2, 5, 10, and 20 nM. After 48 h of treatment, cell viability was measured using the Cell Counting Kit-8 (CCK-8; Biosharp, Hefei, China) assay according to the manufacturer’s instructions. Absorbance was measured using a microplate reader (Bio-Rad Laboratories, Hercules, CA, USA), and viability was normalized to the control group. Dose–response curves were generated using GraphPad Prism software 8.0.

### 4.6. Apoptosis Assay

For the cell apoptosis assay, colorectal cancer cells were treated with IMMU-132 for 48 h. Cells were then harvested, washed with cold PBS, and stained using an Annexin V-APC/propidium iodide staining kit (BD Biosciences, San Jose, CA, USA). After a 15 min incubation at room temperature in the dark, stained cells were analyzed by flow cytometry using a fluorescence-activated cell sorting instrument (BD Biosciences, San Jose, CA, USA). Apoptotic cells were quantified according to Annexin V and propidium iodide positivity. All experiments were performed in triplicate.

### 4.7. Colony Formation Assay

For colony formation assays, cells were seeded in 6-well plates with three replicates per group and treated with IMMU-132 or PBS as indicated. After treatment, cells were cultured in complete medium at 37 °C with 5% CO_2_ until visible colonies formed. Colonies were fixed with methanol, stained with crystal violet, photographed, and counted.

### 4.8. Western Blot Analysis

Cells were washed with PBS and lysed on ice for 30 min in radioimmunoprecipitation assay lysis buffer (P0013B, Beyotime, Haimen, Nantong, China) supplemented with protease and phosphatase inhibitors (P1045, Beyotime). Cell lysates were centrifuged at 12,000× *g* for 10 min at 4 °C, and the supernatants were collected. Protein concentrations were determined using the Pierce BCA Protein Assay Kit (23227, Thermo Fisher Scientific, Waltham, MA, USA). Equal amounts of total protein were separated by SDS-PAGE and transferred to membranes. After blocking, membranes were incubated overnight at 4 °C with the indicated primary antibodies, including anti-TROP2 (90540S, Cell Signaling Technology, Beverly, MA, USA) and anti-β-actin (4967S, Cell Signaling Technology). The membranes were then incubated with appropriate HRP-linked secondary antibodies at room temperature. β-actin was used as the loading control.

### 4.9. In Vivo Therapeutic Study

All animal experiments were performed in accordance with institutional guidelines and were approved by the Institutional Animal Care and Use Committee of Sun Yat-sen University Cancer Center (approval number: L102012025010G, Guangzhou, China; approved on 1 July 2023). Mice were housed under specific-pathogen-free conditions. Sample sizes for animal experiments were determined based on the exploratory nature of this preclinical efficacy study, previous experience with similar in vivo models, feasibility, and ethical considerations to minimize animal use, in accordance with ARRIVE guidelines [29]. Body weight and general clinical conditions, including activity, food intake, hair condition, and death, were monitored throughout treatment.

For the subcutaneous xenograft model, female BALB/c nude mice aged 4–5 weeks were purchased from Guangdong Medical Laboratory Animal Center (Foshan, China). DLD1 cells with stable human TROP2 overexpression (DLD1-hTROP2-OE) or corresponding negative-control cells (DLD1-NE) were suspended in PBS, and 2 × 10^6^ cells in 100 μL PBS were subcutaneously injected into the right axillary region of each mouse. Tumor volume was measured every three days using calipers and calculated as length × width^2^ × 1/2. When tumor volume reached approximately 100 mm^3^, tumor-bearing mice were randomly allocated to receive PBS, irinotecan, or IMMU-132, with six mice per group. IMMU-132 and irinotecan were administered by tail vein injection at 25 mg/kg and 0.57 mg/kg, respectively, once every three days for six cycles. Irinotecan was included as an exploratory SN-38-related reference control under the dosing regimen used in this study; this comparison was not intended to model conventional irinotecan exposure or to establish clinical superiority over irinotecan-based chemotherapy. At the end of treatment, mice were euthanized, and tumors were excised for gross imaging, hematoxylin and eosin staining, and immunohistochemistry.

For the syngeneic intrasplenic liver metastasis model, female C57BL/6J mice aged 5–6 weeks were purchased from Guangdong Medical Laboratory Animal Center (Foshan, China). Luciferase-expressing MC38-NE or MC38-hTROP2-OE cells were suspended in PBS, and 1 × 10^6^ cells in 100 μL PBS were injected into the spleen of each mouse. Two days after intrasplenic injection, liver tumor establishment was monitored using an In Vivo Imaging System (IVIS; PerkinElmer, Waltham, MA, USA). Mice bearing MC38-NE or MC38-hTROP2-OE liver metastases were then randomly allocated to receive PBS, irinotecan, or IMMU-132 using the same dosing regimen described for the subcutaneous xenograft model, with five mice per group. Mice were euthanized on day 17 after intrasplenic injection. Livers were harvested, photographed, weighed, and processed for histological evaluation, and liver metastatic foci were counted at the endpoint.

### 4.10. Immunohistochemistry

Immunohistochemistry (IHC) was performed using paraffin-embedded tumor and liver specimens from the in vivo therapeutic study. Tissue sections were deparaffinized, rehydrated, subjected to antigen retrieval, blocked, and incubated with an anti-Ki-67 antibody (Abcam, Cambridge, UK; ab16667). Staining was visualized using a chromogenic detection system, and sections were counterstained with hematoxylin. Representative images were acquired using a digital slide scanner (Ningbo Jiangfeng Bio-Information Technology Co., Ltd., Ningbo, China). Ki-67-positive tumor cells were quantified in randomly selected representative microscopic fields, avoiding necrotic areas where feasible, by investigators blinded to treatment allocation, and the percentage of Ki-67-positive cells was calculated.

### 4.11. Patient-Derived Organoid Culture and Drug-Response Assay

Fresh primary tumor or liver metastatic tumor tissues were obtained from patients with colorectal liver metastasis who had received first-line systemic therapy and underwent surgical resection at Sun Yat-sen University Cancer Center. Written informed consent was obtained from all patients. The research protocol was approved by the Research Ethics Committee of Sun Yat-sen University Cancer Center (approval number: G2023-029-01; approved on 5 August 2022). All samples were confirmed by pathological assessment. A total of 11 PDOs from 9 patients were included in the IMMU-132 drug-response analysis, comprising 7 liver metastasis-derived PDOs and 4 primary tumor-derived PDOs, including two matched primary tumor and liver metastasis PDO pairs from patients P007 and P030.

Fresh tumor tissues were washed in Hank’s balanced salt solution and minced into small fragments. Tumor fragments were suspended in DMEM/F12 medium (Gibco, Carlsbad, CA, USA) supplemented with the required additives according to the colorectal cancer organoid culture protocol. After repeated trituration and centrifugation, the cell pellet was resuspended in colorectal cancer organoid culture medium (CRC-OCM; Accuroid, Guangzhou, China), mixed with Matrigel (Corning, Corning, NY, USA), and plated as three-dimensional droplets. After gelation, CRC-OCM was added and refreshed regularly. Established organoids were expanded for histological validation and drug-response assays. Representative organoids were fixed, embedded, sectioned, and stained with hematoxylin and eosin.

For drug-response assays, organoids were digested into small fragments or cell clusters and seeded in Matrigel in multi-well plates. After gelling, CRC-OCM was added and then replaced with medium containing serial dilutions of IMMU-132 at concentrations of 400, 100, 25, 6.25, 1.5625, 0.390625, and 0.0976563 μg/mL. After 72 h of treatment, organoid viability was measured using the CellTiter-Glo luminescent cell viability assay (Promega, Madison, WI, USA) according to the manufacturer’s instructions. Luminescence was detected using a chemiluminescent microplate reader (BMG LABTECH, Ortenberg, Germany). Viability was normalized to the control group. Bright-field images were acquired during treatment to document morphological changes.

Dose–response curves were generated for each PDO. To summarize drug response across samples, the normalized area under the dose–response curve (normalized AUC) was calculated over the tested concentration range after normalization to the control condition. Lower normalized AUC values indicated stronger drug-induced inhibition. Normalized AUC values were compared between liver metastasis-derived PDOs and primary tumor-derived PDOs in an exploratory manner. For matched PDO pairs, primary tumor-derived and liver metastasis-derived PDOs from the same patient were compared descriptively.

### 4.12. RNA Sequencing and Transcriptomic Analysis

Total RNA was isolated from DLD1 cells treated with IMMU-132 or vehicle control using Magzol Reagent (Magen, Guangzhou, China) according to the manufacturer’s protocol. RNA quantity and integrity were assessed using the K5500 system (Beijing Kaiao, Beijing, China) and the Agilent 2200 TapeStation (Agilent Technologies, Santa Clara, CA, USA). mRNA was enriched using the NEBNext Poly(A) mRNA Magnetic Isolation Module (New England Biolabs, Ipswich, MA, USA), fragmented to approximately 200 bp, and used for first- and second-strand cDNA synthesis, adaptor ligation, and low-cycle amplification using the NEBNext Ultra RNA Library Prep Kit for Illumina. Purified libraries were evaluated using the Agilent 2200 TapeStation and Qubit (Thermo Fisher Scientific, Waltham, MA, USA), and sequenced on an Illumina platform (Illumina, San Diego, CA, USA) with paired-end 150 bp reads by Ribobio Co., Ltd. (Guangzhou, China). Clean reads were obtained after removing adaptor-contaminated, poly-N, and low-quality reads. HISAT2 (version 2.2.1) was used to align clean reads to the human reference genome hg38, and HTSeq (version 2.0.5) was used to generate read counts for each gene model. Differential expression analysis was performed using edgeR (version 4.4.2) with Benjamini–Hochberg correction. Differentially expressed genes were defined by fold change > 2 and *p* < 0.05 and were used for functional enrichment analyses.

### 4.13. γ-H2AX Immunofluorescence

For γ-H2AX immunofluorescence analysis, control and TROP2-knockdown colorectal cancer cells were treated with IMMU-132 or vehicle as indicated. Cells were fixed with 4% paraformaldehyde, permeabilized with Triton X-100, blocked with bovine serum albumin, and incubated with an anti-phospho-H2A.X antibody (Servicebio, Wuhan, China; GB111841-100) overnight at 4 °C. After washing, cells were incubated with an appropriate fluorescent secondary antibody and counterstained with DAPI. Representative images were acquired using a Nikon confocal laser scanning microscope (Nikon Corporation, Tokyo, Japan) under comparable imaging settings for exploratory visualization of the DNA damage-associated γ-H2AX signal.

### 4.14. Statistical Analysis

All data are presented as the mean ± SEM unless otherwise indicated. Statistical analyses were performed using GraphPad Prism (version 8.0; GraphPad Software, San Diego, CA, USA) and R. In vitro assays were performed with at least three independent biological experiments unless otherwise indicated. Data distribution was assessed when sample size permitted. Comparisons between two groups were performed using two-tailed Student’s *t*-test for approximately normally distributed data or the Mann–Whitney U test for non-normally distributed data. Comparisons among multiple groups were performed using one-way analysis of variance followed by appropriate post hoc tests or non-parametric alternatives when applicable. Tumor growth curves were analyzed using repeated-measures or two-way analysis of variance. Survival curves were compared using the log-rank test. For RNA-seq analysis, Benjamini–Hochberg correction was applied when applicable. Sample-level TACSTD2-positive proportions in the single-cell analysis were presented descriptively because of the limited number of liver metastasis samples. *p* values < 0.05 were considered statistically significant. *p* values are shown in the figures and/or legends with asterisks. *, *p* < 0.05; **, *p* < 0.01; ***, *p* < 0.001; ****, *p* < 0.0001.

## 5. Conclusions

In conclusion, this study systematically evaluated the preclinical pharmacological activity of sacituzumab govitecan/IMMU-132 in colorectal liver metastasis using in vitro cell models, in vivo mouse models, and patient-derived organoids. By integrating exploratory single-cell transcriptomic reanalysis, functional TROP2 modulation, in vivo liver metastasis modeling, patient-derived organoids, and molecular response profiling, we showed that TACSTD2/TROP2-positive epithelial-associated cells may represent a targetable tumor-associated population in CRLM. IMMU-132 exhibited antitumor activity in TROP2-expressing colorectal cancer models, suppressed liver metastatic burden, and showed exploratory activity in liver metastasis-derived PDOs. Considering that all PDOs in this study were derived from CRLM patients after first-line therapy, these findings provide a preclinical pharmacological rationale for further evaluation of TROP2-directed SN-38 delivery in biomarker-annotated CRLM models after first-line systemic therapy, and may inform future translational and drug-development studies.

## Data Availability

The public single-cell RNA-sequencing dataset analyzed in this study is available from the Gene Expression Omnibus under accession number GSE315534. Other data supporting the findings of this study, including code used for selected analyses and figure generation, are available from the corresponding author upon reasonable request.

## Supplementary Materials

The following supporting information can be downloaded at: https://www.mdpi.com/article/10.3390/ph19081163/s1, Figure S1: Major cell-type annotation, epithelial-associated cell selection, and patient/sample-level TACSTD2/TROP2-positive epithelial-associated cell proportions in the GSE315534 single-cell atlas; Figure S2: Individual IMMU-132 dose–response curves of patient-derived organoids; Figure S3: Exploratory comparison of IMMU-132 and irinotecan responses in patient-derived organoids; Table S1: Clinicopathological and molecular characteristics of the CRLM PDO cohort.

## Abbreviations

The following abbreviations are used in this manuscript: ADC, antibody–drug conjugate; AUC, area under the curve; CRC, colorectal cancer; CRLM, colorectal liver metastasis; DAPI, 4′,6-diamidino-2-phenylindole; GO, Gene Ontology; hTROP2, human TROP2; IHC, immunohistochemistry; IVIS, In Vivo Imaging System; KEGG, Kyoto Encyclopedia of Genes and Genomes; LM, liver metastasis; NC, non-targeting shRNA negative control; NE, empty-vector negative control; OE, overexpression; PBS, phosphate-buffered saline; PDO, patient-derived organoid; PI, propidium iodide; scRNA-seq, single-cell RNA sequencing; SG, sacituzumab govitecan; shNC, short hairpin negative control; shTROP2, short hairpin RNA targeting TROP2; TACSTD2, tumor-associated calcium signal transducer 2; TROP2, trophoblast cell-surface antigen 2; UMAP, Uniform Manifold Approximation and Projection.
