# Supplementary material for: Preclinical Pharmacological Evaluation of Sacituzumab Govitecan (IMMU-132) in TROP2-Positive Colorectal Liver Metastasis Models
Source: Pharmaceuticals (Basel). 2026 Jul 25;19(8):1163. doi: 10.3390/ph19081163 (PMC13516231; doi:10.3390/ph19081163)
Supplement: Supplementary file 1 [file pharmaceuticals-19-01163-s001.zip › pharmaceuticals-4395656-supplementary.pdf]

Supplementary Materials

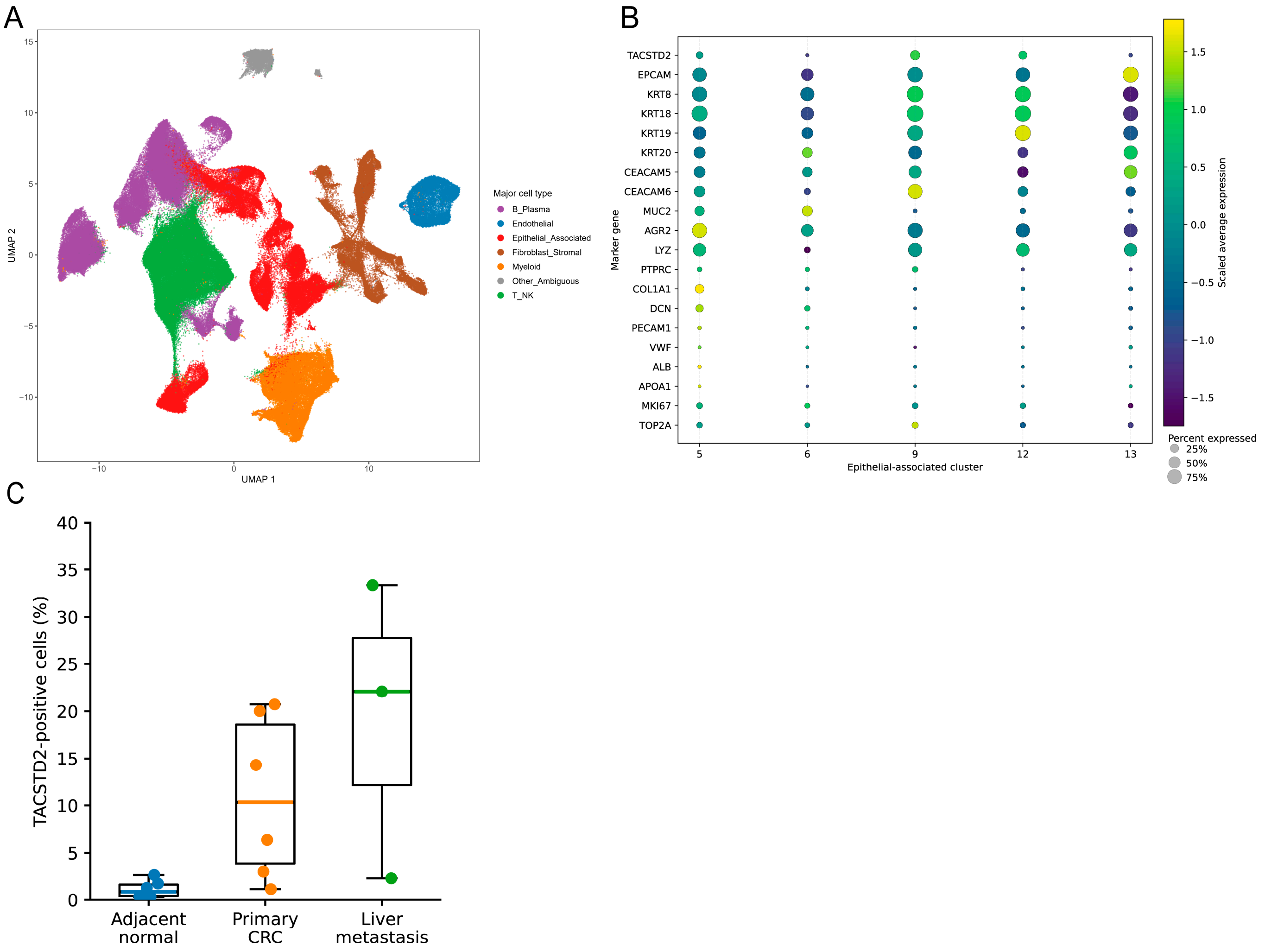

Figure S1. Major cell-type annotation, epithelial-associated cell selection, and patient/sample-level TACSTD2/TROP2-positive epithelial-associated cell proportions in the GSE315534 single-cell atlas.

A. UMAP visualization of the GSE315534 whole-tumor scRNA-seq atlas colored by major cell type, including B/plasma cells, endothelial cells, epithelial-associated cells, fibroblast/stromal cells, myeloid cells, T/NK cells, and other ambiguous cells.

B. Dot plot showing the expression of representative marker genes across epithelial-associated clusters. Clusters 5, 6, 9, 12, and 13 belonged to the epithelial-associated compartment and expressed canonical epithelial markers, including EPCAM and multiple keratins, with limited expression of immune, stromal, endothelial, and hepatocyte markers. Dot size indicates the percentage of cells expressing each marker, and color indicates scaled average expression.

C. Box-and-dot plot showing the patient/sample-level proportion of TACSTD2-positive epithelial-associated cells across adjacent normal tissues, primary colorectal tumors, and liver metastases. Each dot represents one patient/sample. Adjacent normal tissues (n = 6), primary colorectal tumors (n = 6), and liver metastases (n = 3) are shown. Boxes indicate the interquartile range, horizontal lines indicate the median, and whiskers indicate the data range. Because only three liver metastasis samples were available, this analysis was presented descriptively without formal patient-level statistical inference.

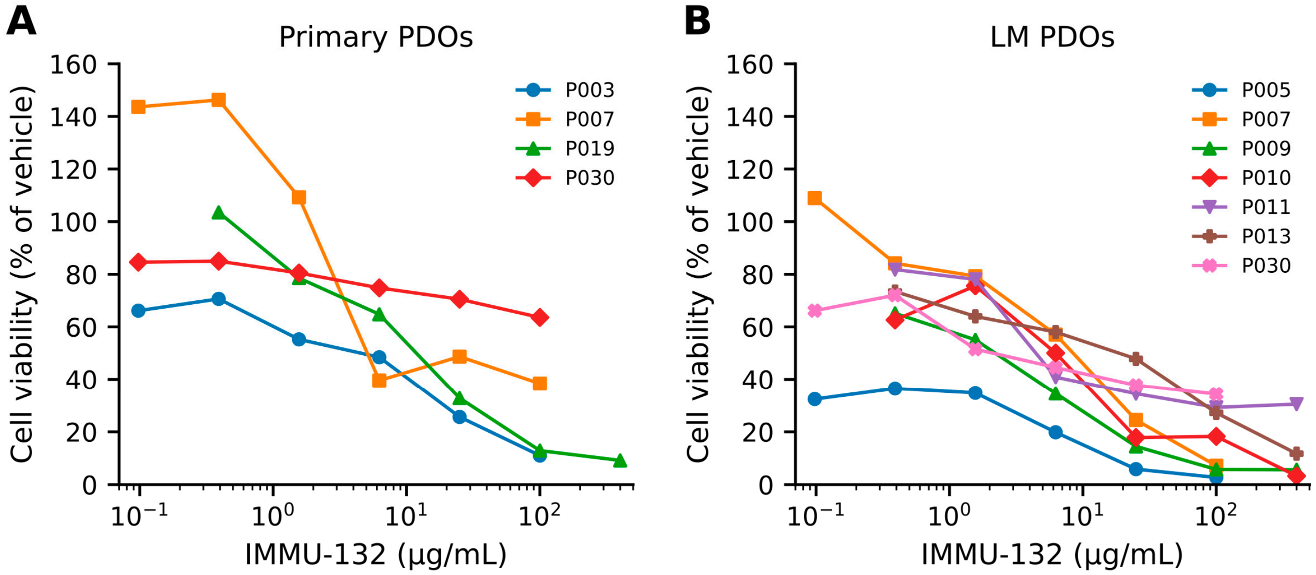

Figure S2. Individual IMMU-132 dose–response curves of patient-derived organoids.

A. Individual dose-response curves of primary tumor-derived PDOs treated with serial concentrations of IMMU-132. Cell viability was determined by CellTiter-Glo assay and normalized to the vehicle-treated control group.

B. Individual dose-response curves of liver metastasis-derived PDOs treated with serial concentrations of IMMU-132. Cell viability was determined by CellTiter-Glo assay and normalized to the vehicle-treated control group.

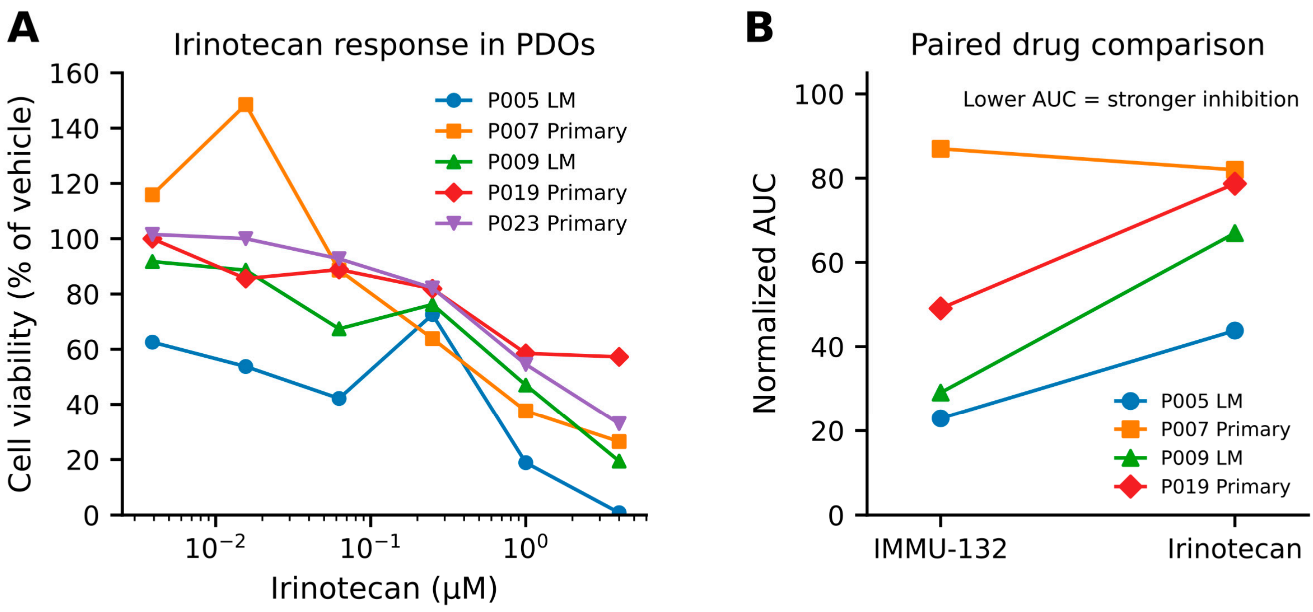

Figure S3. Exploratory comparison of IMMU-132 and irinotecan responses in patient-derived organoids.

A. Individual irinotecan dose-response curves in PDOs tested with irinotecan. Cell viability was determined by CellTiter-Glo assay and normalized to the vehicle-treated control group.

B. Paired comparison of normalized AUC values between IMMU-132 and irinotecan in PDOs tested with both drugs. Each connected line represents one PDO sample. Lower normalized AUC indicates stronger drug-induced inhibition.

Table S1. Clinicopathological and molecular characteristics of the CRLM PDO cohort.

De-identified ID	Primary tumor location	KRAS mutation status	BRAF mutation status	First-line chemotherapy regimen	Targeted agent	Microsatellite status
P003	Ileocecal region	WT	WT	XELOX	Bevacizumab	MSS
P005	Rectum	WT	WT	XELOX	Cetuximab	MSS
P007	Ileocecal region	G12D	WT	XELOX	Bevacizumab	MSS
P009	Rectum	WT	WT	XELOX	Cetuximab	MSS
P010	Hepatic flexure	WT	WT	XELOX	Cetuximab	MSS
P011	Ascending colon	WT	WT	XELOX	Cetuximab	MSS
P013	Descending colon	WT	WT	XELOX	Cetuximab	MSS
P019	Rectum	G12D	WT	XELOX	Bevacizumab	MSS
P030	Ascending colon	G12V	WT	XELOX	Bevacizumab	MSS

Abbreviations: BRAF, B-Raf proto-oncogene; KRAS, Kirsten rat sarcoma viral oncogene homolog; MSS, microsatellite stable; WT, wild-type; XELOX, capecitabine plus oxaliplatin.
